# Caged mitochondrial uncouplers that are released in response to hydrogen peroxide

**DOI:** 10.1016/j.tet.2010.01.103

**Published:** 2010-03-27

**Authors:** Caroline Quin, Linsey Robertson, Stephen J. McQuaker, Nicholas C. Price, Martin D. Brand, Richard C. Hartley

**Affiliations:** a Buck Institute for Age Research, 8001 Redwood Boulevard, Novato, CA 94945, USA; b Centre for the Chemical Research of Ageing, WestCHEM Department of Chemistry, University of Glasgow, Glasgow G12 8QQ, United Kingdom; c Faculty of Biomedical and Life Sciences, Joseph Black Building, University of Glasgow, Glasgow G12 8QQ, United Kingdom

**Keywords:** Mitochondria, Caged, Uncoupler, Boronate, Hydrogen peroxide, Ageing, Oxidative stress, ROS

## Abstract

Caged versions of the most common mitochondrial uncouplers (proton translocators) have been prepared that sense the reactive oxygen species (ROS) hydrogen peroxide to release the uncouplers 2,4-dinitrophenol (DNP) and carbonylcyanide *p*-trifluoromethoxyphenylhydrazone (FCCP) from caged states with second order rate constants of 10 (±0.8) M^−1^ s^−1^ and 64.8 (±0.6) M^−1^ s^−1^, respectively. The trigger mechanism involves conversion of an arylboronate into a phenol followed by fragmentation. Hydrogen peroxide-activated uncouplers may be useful for studying the biological process of ageing.

## Graphical abstract

**Figure:**
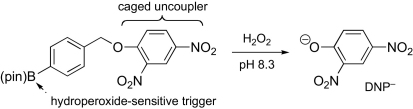

## 1 Introduction

Across the world, life expectancy is steadily increasing while the birth rate is dropping, leading to an ageing population.^1^ Since age is the primary risk factor for many diseases,^2^ understanding the mechanisms of ageing may allow us to reduce significantly the burden of disease and increase human healthspan. Arguably the most convincing theory of ageing is the mitochondrial free radical theory,^3^ which postulates that the generation of reactive oxygen species (ROS) in mitochondria is an inevitable consequence of oxidative ATP production, and that the ROS are the primary cause of the macromolecular damage that accumulates to limit lifespan. However, it is agreed that the theory's explanation of ageing is incomplete.^4^ For these reasons, fluorescent probes have been developed for detecting ROS,^5^ and potential therapeutic antioxidants, particularly those which target the mitochondrion,^6,7^ are of increasing interest.

Here we introduce the new concept of functional molecules that are designed to sense ROS and respond by shutting down the biological processes leading to their production, rather than interacting directly with the ROS like conventional antioxidants. Importantly, the functional molecules would moderate such processes only when they were leading to the production of excess ROS that could not be effectively managed by the endogenous antioxidant system. Their selective and responsive nature should make them useful chemical biological tools for understanding the effects of ROS-generating processes on cellular damage and on lifespan. The advantage of using chemical biology, rather than molecular biology, would be that the functional molecules could be used in any cell line or organism in a dose-dependent and reversible way. Here we demonstrate the chemical reactivity of the first prototypes.

**Scheme 1:**
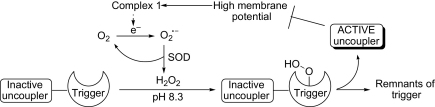
Proposed mode of action of caged uncouplers.

The electron transport chain in the mitochondrial inner membrane pumps protons out of the mitochondrial matrix to generate an electrochemical gradient of protons. Protons flowing back through ATP synthase, then convert the energy of the proton motive force into chemical energy in the form of ATP. There is evidence that when the potential across this membrane rises there is a substantial increase in ROS and many more evade capture by the defence system.^8,9^ Production of excess ROS involves generation of superoxide on the inside of the mitochondrial inner membrane by electron-leakage from complex I and complex III of the electron transport chain.^8,10^ Superoxide dismutase (SOD) rapidly converts this superoxide into hydrogen peroxide, which is uncharged at physiological pH and diffuses away from the matrix of the mitochondrion. In the event of the hydrogen peroxide coming into contact with iron(II) ions, hydroxyl radicals are produced that react with biomolecules at rates close to that of diffusion causing damage. Our prototype functional molecules are designed to sense hydrogen peroxide and respond by releasing a proton-translocator (uncoupler) that will allow protons to flow back across the mitochondrial inner membrane without passing through ATP synthase (uncoupling the electron transport chain from ATP production),^11^ so reducing the membrane potential and stopping ROS production (Scheme 1). Here there is an interesting parallel with uncoupling proteins, UCP2 and UCP3, which are postulated to cause mild uncoupling in response to oxidative stress as part of the natural antioxidant defence system;^10,12^ although this remains controversial.^13^

**Figure 1:**
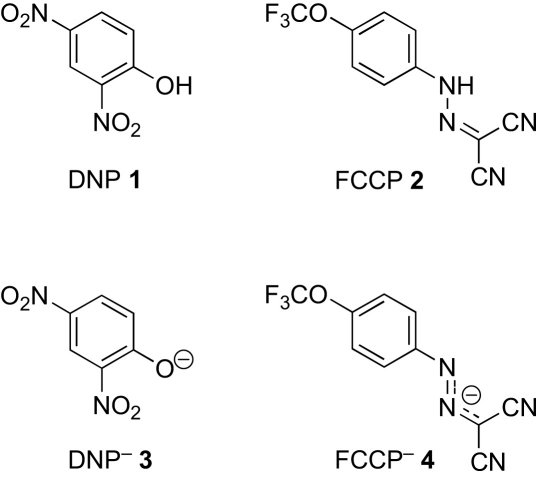
Uncouplers in their protonated and unprotonated forms.

**Scheme 2:**
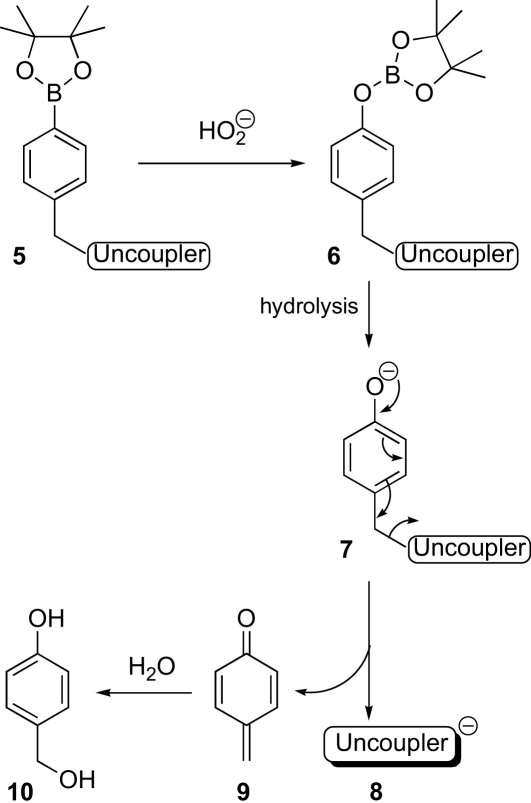
Reaction between caged uncouplers and hydrogen peroxide.

Arylboronate esters react with hydrogen peroxide in mildly alkaline media to give phenols (e.g., at pH 8.3, which is the approximate pH of the matrix of active mitochondria), and this has been used to make a range of fluorescent sensors that are sensitive to, and selective for, hydrogen peroxide.^5,14,15^ One mode of action involves the conversion of an arylboronate into a phenol, which fragments to release a fluorescent molecule.^15^ We aimed to use this reaction to release the most commonly employed mitochondrial uncouplers, dinitrophenol (DNP)^11,16,17^ **1** and carbonylcyanide *p*-trifluoromethoxyphenylhydrazone (FCCP) **2** (Fig. 1).^11,18,19^ These are mildly acidic compounds (p*K*_a_ 4.1^20^ and 5.8,^21^ respectively) that are deprotonated in the mitochondrial matrix to form lipophilic anions DNP^−^ **3** and FCCP^−^ **4**, which can cross the inner mitochondrial membrane, pick up a proton, and return. Thus, arylboronate **5**, bearing a caged uncoupler, should react with the hydroperoxide ion in the alkaline medium of the mitochondrial matrix to give a borate ester intermediate **6**. Hydrolysis to give the phenoxide ion **7** would then be followed by fragmentation to give the active uncoupler **8** and the quinone methide side product **9**, which would be trapped rapidly by hydroxide or water to give 4-hydroxybenzyl alcohol **10** (Scheme 2).^22^

**Scheme 3:**
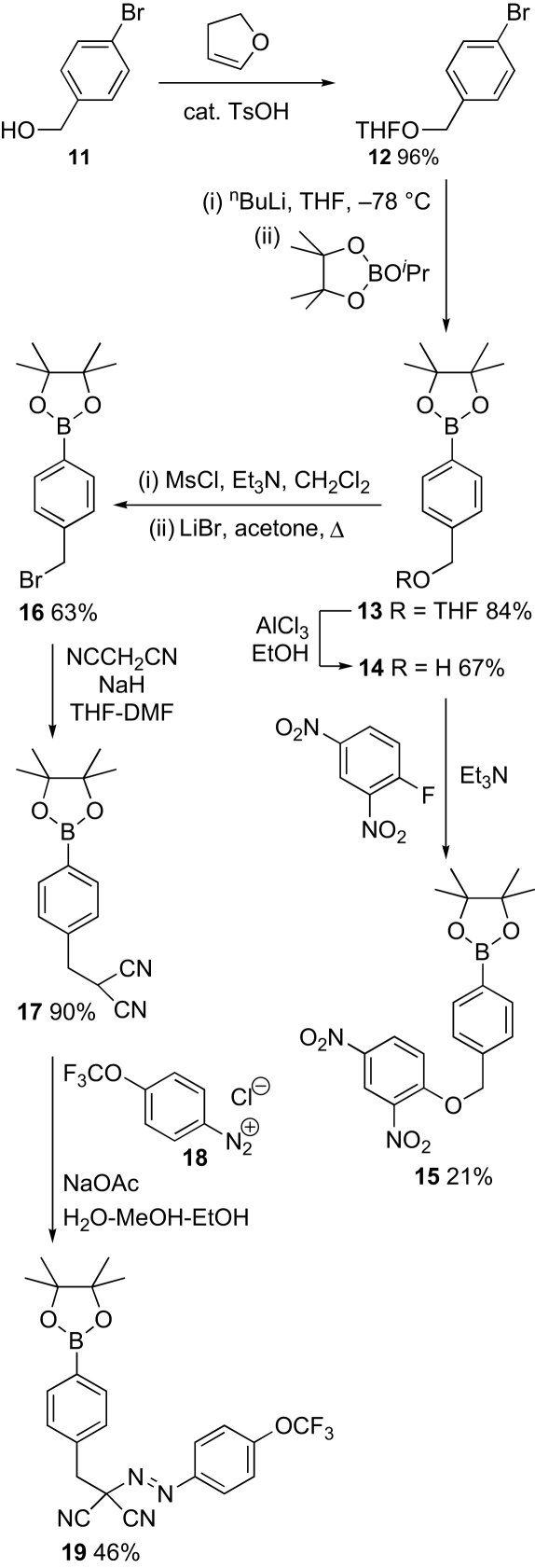
Synthesis of caged uncouplers **15** and **19**.

Our first targets, caged uncouplers **15** and **19** were synthesized as shown (Scheme 3). 4-Bromobenzylic alcohol **11** was protected as the THF acetal **12**. Lithiation–boronation gave arylboronate **13**. This was deprotected to give alcohol **14**, which was used to displace fluoride from 2,4-dinitro-1-fluorobenzene to yield caged uncoupler **15**. Alcohol **14** was converted into the bromide **16** by the literature method,^23^ reaction with deprotonated malononitrile gave arylboronate **17**. This was then coupled with diazonium salt **18**, freshly generated from 4-trifluoromethoxyaniline, to give caged uncoupler **19**.

**Figure 2:**
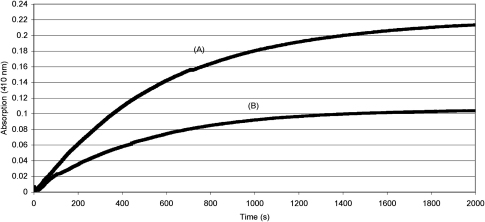
Hydrogen peroxide triggered release of DNP/DNP^−^ from a 200 μM solution of caged uncoupler **15** in 1:1 DMF/aqueous NaHCO_3_ monitored by absorbance at 410 nm. Initial concentrations of hydrogen peroxide were 20 μM for line (A) and 10 μM for line (B).

The reaction between the caged uncouplers **15** and **19** and alkaline hydrogen peroxide was studied under *pseudo*-first order conditions. One millilitre of a 200 μM solution of the caged uncoupler **15** in a 1:1 mixture of DMF and 0.14 M aqueous sodium bicarbonate (pH 8.3) was treated at 37 °C with 20 μM aqueous hydrogen peroxide and the absorption monitored at 410 nm (the absorption maximum of DNP^−^ **3** under these conditions). The average of three runs is presented in Figure 2. This gave a Δ*A*_lim_ of 0.220 (±0.006), corresponding to 10.7 μM DNP^−^ **3** released or a yield of 54% relative to the hydrogen peroxide added. The average apparent first order rate constant was 1.85×10^−3^ s^−1^, giving a second order rate constant *k*_DNP_ of 9.3 M^−1^ s^−1^. Repeating the experiment with 10 μM hydrogen peroxide gave a Δ*A*_lim_ of 0.122 (±0.01), corresponding to 5.96 μM DNP^−^ **3** released or a yield of 60% relative to the hydrogen peroxide added. The average apparent first order rate constant was 2.16×10^−3^ s^−1^, giving a second order rate constant *k*_DNP_ of 10.8 M^−1^ s^−1^. The overall value for *k*_DNP_ is therefore 10 (±0.8) M^−1^ s^−1^ and a 57 (±3)% yield of DNP^−^ **3** was obtained with respect to the hydrogen peroxide used. The stoichiometry of hydrogen peroxide to caged uncoupler **15** is clearly greater than 1:1. A plausible explanation for this is that the *para*-quinone methide produced by fragmentation of the probe is predominantly trapped by hydrogen peroxide rather than water.

Similar experiments with caged uncoupler **19** gave good *pseudo*-first order kinetics, a calculated second order rate constant *k*_FCCP_ of 64.8 (±0.6) M^−1^ s^−1^ at pH 8.3 and a 58 (±2)% yield of FCCP^−^ **4** relative to the hydrogen peroxide used. For comparison, the second order rate constant for reaction between glutathione and hydrogen peroxide at 37 °C is only 0.87 M^−1^ s^−1^, albeit at pH 7.4. Thus, we have demonstrated that caged uncouplers **15** and **19** react with alkaline hydrogen peroxide to release uncouplers at a biologically appropriate rate.

In summary, we have prepared the first examples of hydrogen peroxide-activated caged uncouplers and demonstrated that they release the two most common uncoupling agents, DNP^−^ **3** and FCCP^−^ **4**, at a useful rate. The next stage will be to target these caged uncouplers to mitochondria and test the mitochondrial free radical theory of ageing.

## 2 Experimental

### 2.1 Synthesis

All reactions under an inert atmosphere were carried out using oven dried or flame-dried glassware. Solutions were added via syringe. Tetrahydrofuran and dichloromethane were dried where necessary using a solvent drying system, Puresolv™, in which solvent is pushed from its storage container under low nitrogen pressure through two stainless steel columns containing activated alumina and copper. Acetone was dried over 4 Å molecular sieves. Triethylamine was dried by distillation over calcium hydride and stored over potassium hydroxide. Reagents, including DNP **1**, were obtained from commercial suppliers and used without further purification unless otherwise stated. ^1^H, ^13^C and ^19^F NMR spectra were obtained on a Bruker DPX/400 spectrometer operating at 400, 100 and 376 MHz, respectively. All coupling constants are measured in hertz. DEPT was used to assign the signals in the ^13^C NMR spectra as C, CH, CH_2_ or CH_3_. Mass spectra (MS) were recorded on a Jeol JMS700 (MStation) spectrometer. Infrared (IR) spectra were obtained on a Shimadzu FTIR-8400S spectrometer using attenuated total reflectance (ATR) so that the IR spectrum of the compound (solid or liquid) could be directly detected (thin layer) without any sample preparation.

### 2.2 Carbonyl cyanide 4-(trifluoromethoxy)phenylhydrazone (FCCP) **2**

A solution of NaNO_2_ (350 mg, 5.0 mmol) in water (5 mL) was cooled to 0 °C. This solution was added dropwise to a stirred solution of 4-trifluoromethoxyaniline (886 mg, 5.0 mmol) in aqueous hydrochloric acid (1.6 M, 35 mL) at 0 °C. The mixture was stirred at 0 °C for 5 min and then added dropwise into a stirred solution of malononitrile (0.48 mL, 7.5 mmol) and NaOAc (12.5 g, 152 mmol) in water (50 mL). A yellow precipitate formed was collected by filtration and was washed with ice-cold water. The precipitate was dissolved in Et_2_O, dried over MgSO_4_ and the solvent removed under reduced pressure. Recrystallization from EtOAc/hexane gave FCCP **2** as a yellow amorphous solid (951 mg, 75%). Mp 145–148 °C (decomp.). *ν*_max_ (ATR): 3066 (CH), 2226 (CN), 1618 (Ar) cm^−1^. *δ*_H_ (CDCl_3_, 400 MHz): 7.53 (2H, d, *J*=9.2 Hz, H-2 and H-6), 7.20 (2H, m, H-3 and H-5). *δ*_C_ (CDCl_3_, 100 MHz): 147.89 (C), 141.66 (C), 123.48 (CH), 121.91 (CF_3_, q, *J*=254.1 Hz), 118.75 (CH), 114.47 (C), 109.85 (C), 87.57 (C). ^1^H and ^13^C NMR in good agreement with literature.^24^

### 2.3 2-(4′-Bromobenzyloxy)tetrahydrofuran **12**

A solution of 4-bromobenzyl alcohol **11** (13.00 g, 69.5 mmol), 2,3-dihydrofuran (6.3 mL, 83.5 mmol) and *para*-toluene sulfonic acid monohydrate (20 mg, 0.1 mmol) in anhydrous DCM (50 mL) was stirred at rt for 1.5 h. The reaction mixture was then washed with H_2_O (2×100 mL) and saturated aqueous NaHCO_3_ (100 mL) and the organic phase dried over MgSO_4_ and concentrated under reduced pressure to afford the acetal **12** as a yellow oil (17.17 g, 96%). *ν*_max_ (ATR): 2982 (CH), 2951 (CH), 2882 (CH), 1487 (Ar) cm^−1^. *δ*_H_ (CDCl_3_, 400 MHz): 7.45 (2H, d, *J*=8.3 Hz, H-3′ and H-5′), 7.20 (2H, d, *J*=8.3 Hz, H-2′ and H-6′), 5.19 (1H, dd, *J*=2.6 and 3.7 Hz, H-2), 4.65 (1H, d, *J*=12.4 Hz, ArC*H*^A^H^B^), 4.42 (1H, d, *J*=12.4 Hz, ArCH^A^*H*^B^), 3.95–3.84 (2H, m, 2×H-5), 2.07–1.80 (4H, m, 2×H-3 and 2×H-4). *δ*_C_ (CDCl_3_, 100 MHz): 137.44 (C), 131.41 (CH), 129.43 (CH), 121.29 (C), 103.17 (CH), 67.97 (CH_2_), 67.08 (CH_2_), 32.34 (CH_2_), 23.44 (CH_2_). LRMS (EI^+^): 258 [M^+^^•^, (^81^Br), 13%], 256 [M^+^^•^, (^79^Br), 13], 171 (^81^BrC_6_H_4_CH_2_^+^, 100), 169 (^79^BrC_6_H_4_CH_2_^+^, 100), 71 (C_4_H_7_O^+^, 100). HRMS: 258.0079 and 256.0099. C_11_H_13_^81^BrO_2_ requires M^+^^•^, 258.0085 and C_11_H_13_^79^BrO_2_ requires M^+^^•^, 256.0097.

### 2.4 2-[4′-(4″,4″,5″,5″-Tetramethyl-1″,3″,2″-dioxaborolan-2″-yl)benzyloxy]tetrahydrofuran **13**

A solution of bromide **12** (12.00 g, 46.7 mmol) in anhydrous THF (130 mL) was cooled to −78 °C and degassed with argon for 15 min. Under argon, *^n^*BuLi (1.68 M in hexanes, 31.7 mL, 56.0 mmol) was added dropwise over 3 h and the mixture allowed to stir for 15 min. 2-Isopropoxy-4,4,5,5-tetramethyl-1,3,2-dioxaborolane (11.4 mL, 56.0 mmol) was added dropwise and the resulting solution allowed to stir for 2 h. The reaction was quenched with H_2_O (100 mL) and the product extracted with DCM (3×100 mL). The organic layers were combined, washed with brine (100 mL), dried over MgSO_4_ and concentrated under reduced pressure to give a yellow oil. Impurities were removed by Kugelrohr distillation leaving acetal **13** as the residual yellow oil, which solidified on standing (11.87 g, 84%). Mp: 41–44 °C. *ν*_max_ (ATR): 2978 (CH), 2932 (CH), 2882 (CH), 1614 (Ar) cm^−1^. *δ*_H_ (CDCl_3_, 400 MHz): 7.78 (2H, d, *J*=7.7 Hz, H-3′ and H-5′), 7.34 (2H, d, *J*=7.7 Hz, H-4′ and H-6′), 5.20 (1H, dd, *J*=1.2 and 4.2 Hz, H-2′), 4.72 (1H, d, *J*=12.4 Hz, ArC*H*^A^H^B^), 4.50 (1H, d, *J*=12.4 Hz, ArCH^A^*H*^B^), 3.97–3.86 (2H, m, 2×H-5), 2.09–1.83 (4H, m, 2×H-3 and 2×H-4), 1.34 (12H, s, 4×Me). *δ*_C_ (CDCl_3_, 100 MHz): 141.60 (C), 134.86 (CH), 126.99 (CH), 103.09 (CH), 83.73 (C), 68.60 (CH_2_), 67.02 (CH_2_), 32.33 (CH_2_), 24.84 (CH_3_), 23.44 (CH_2_). LRMS (EI^+^): 304 (M^+^^•^, 4%), 217 (75), 83 (100). HRMS: 304.1842. C_17_H_25_^11^BO_4_ requires M^+^^•^, 304.1846.

### 2.5 [4-(4′,4′,5′,5′-Tetramethyl-1′,3′,2′-dioxaborolan-2′-yl)phenyl]methanol 14

Aluminium trichloride (40 mg, 0.30 mmol) was added to a stirred solution of acetal **13** (1.00 g, 3.29 mmol) in anhydrous EtOH (10 mL). The mixture was stirred overnight at rt and then concentrated under reduced pressure. The mixture was dissolved in Et_2_O and undissolved solid was removed by filtration. The filtrate was evaporated under reduced pressure and redissolved in anhydrous EtOH (10 mL). Aluminium trichloride (30 mg, 0.22 mmol) was added and the solution was stirred overnight at rt. The mixture was concentrated under reduced pressure, dissolved in Et_2_O and filtered to remove undissolved solid. The solution was then concentrated under reduced pressure to afford the benzylic alcohol **14** as an oil (517 mg, 67%). *ν*_max_ (ATR): 3312 (OH), 2978 (CH), 2930 (CH), 2880 (CH), 1614 (Ar) cm^−1^. *δ*_H_ (CDCl_3_, 400 MHz): 7.78 (2H, d, *J*=7.6 Hz, H-3 and H-5′), 7.33 (2H, d, *J*=7.7 Hz, H-2 and H-6), 4.66 (2H, s, ArCH_2_), 1.33 (12H, s, 4×Me). *δ*_C_ (CDCl_3_, 100 MHz): 144.07 (C), 134.99 (CH), 126.06 (CH), 83.82 (C), 65.05 (CH_2_), 24.83 (CH_3_). LRMS (EI^+^): 234 [M^+^^•^, 50%], 135 [HOCH_2_C_6_H_4_^11^BOH^+^, 100]. HRMS: 234.1426. C_13_H_19_^11^BO_3_ requires M^+^^•^, 234.1427. ^1^H and ^13^C NMR agree with literature.^23^

### 2.6 2-[4′-(2″,4″-Dinitrophenoxymethyl)phenyl]-4,4,5,5-tetramethyl-1,3,2-dioxaborolane **15**

Benzylic alcohol **14** (200 mg, 0.854 mmol) was mixed with 2,4-dinitrofluorobenzene (0.11 mL, 4.1 mmol) and two drops of anhydrous triethylamine added. The reaction mixture was stirred at rt overnight and then concentrated under reduced pressure. The mixture was triturated with ether and the resulting pale yellow solid recrystallized from EtOAc/hexane to afford caged uncoupler **15** as an amorphous solid (72 mg, 21%). *R_f_* [SiO_2_, petroleum ether/EtOAc (4:1)]: 0.23. Mp: 143–145 °C. *ν*_max_ (ATR): 2981 (CH), 2931 (CH), 2894 (CH), 1604 (Ar), 1521 (NO_2_), 1342 (NO_2_) cm^−1^. *δ*_H_ (CDCl_3_, 400 MHz): 8.74 (1H, d, *J*=2.8 Hz, H-3″), 8.34 (1H, dd, *J*=2.8 and 9.3 Hz, H-5″), 7.85 (2H, d, *J*=8.1 Hz, H-2′ and H-6′), 7.44 (2H, d, *J*=8.1 Hz, H-3′ and H-6′), 7.21 (1H, d, *J*=9.3 Hz, H-6″), 5.39 (2H, s, ArCH_2_), 1.35 (12H, s, 4×Me). *δ*_C_ (CDCl_3_, 100 MHz): 156.21 (C), 140.23 (C), 139.24 (C), 136.90 (C), 135.39 (CH), 128.95 (CH), 126.15 (CH), 121.96 (CH), 114.98 (CH), 84.01 (C), 71.98 (CH_2_), 24.86 (CH_3_). LRMS (CI^+^): 401 [(M+H)^+^, 50%], 219 (100). HRMS: 401.1521. C_19_H_22_^11^BN_2_O_7_ requires (M+H)^+^, 401.1520.

### 2.7 2-(4′-Bromomethylphenyl)-4,4,5,5-tetramethyl-1,3,2-dioxaborolane **16**

Following the procedure of De Filippis et al.,^23^ benzylic alcohol **14** (500 mg, 2.14 mmol) was dissolved in anhydrous DCM (15 mL) and degassed with argon for 15 min. The solution was cooled to 0 °C and anhydrous triethylamine (324 mg, 3.20 mmol) was added. Methanesulfonyl chloride (198 μL, 2.56 mmol) was added and the solution was allowed to stir for 1 h. Organics were washed with H_2_O (3×4 mL), dried over MgSO_4_ and concentrated under reduced pressure. The resultant solid was dissolved in anhydrous acetone (15 mL) and degassed with argon for 15 min. Lithium bromide (1.90 g, 21.4 mmol) was added and the resultant solution was heated under reflux overnight. The solution was concentrated under reduced pressure and the residue dissolved in DCM (15 mL), washed with H_2_O (3×4 mL), dried over MgSO_4_ and concentrated under reduced pressure to yield the target bromide **16** as an amorphous solid (413 mg, 63%). Mp: 76–78 °C (cubes from Et_2_O/hexane. Lit.^23^ 75–77 °C). *ν*_max_ (ATR): 2976 (CH), 2930 (CH), 2872 (CH), 1613 (Ar) cm^−1^. *δ*_H_ (400 MHz, CDCl_3_): 7.79 (2H, d, *J*=7.7 Hz, H-2′ and H-6′), 7.39 (2H, d, *J*=7.7 Hz, H-3′ and H-5′), 4.49 (2H, s, CH_2_), 1.34 (12H, s, 4×Me). *δ*_C_ (100 MHz, CDCl_3_): 140.65 (C), 135.21 (CH), 128.30 (CH), 83.90 (C), 33.33 (CH_2_), 24.84 (CH_3_). LRMS (EI^+^): 298 [M^+^^•^, (^81^Br), 4%], 296 [M^+^^•^, (^79^Br), 4], 217 [M^+^^•^−Br^•^, 100]. HRMS: 298.0549 and 296.0567. C_13_H_18_^11^B^81^BrO_2_ requires M^+^^•^, 298.0563 and C_13_H_18_^11^B^79^BrO_2_ requires M^+^^•^, 296.0583. ^13^C NMR agrees with literature.^23^

### 2.8 2-Cyano-2-[4′-(4″,4″,5″,5″-tetramethyl-1,3,2-dioxaborolan-2-yl)phenyl]propionitrile **17**

A suspension of NaH (80% in mineral oil, 70 mg, 2.3 mmol) in anhydrous THF (16 mL) and anhydrous DMF (1.6 mL) was stirred at rt under argon. A solution of malononitrile (0.12 mL, 2.3 mmol) in anhydrous THF (2 mL) was added dropwise to the solution and hydrogen gas was evolved. The reaction mixture was stirred at rt for 30 min under argon before a solution of bromide **16** (329 mg, 1.11 mmol) in anhydrous THF (4 mL) was added dropwise. The reaction mixture was stirred at 25 °C overnight. Saturated aqueous NH_4_Cl (40 mL) was added to quench the reaction and the mixture was extracted with EtOAc (3×20 mL). The combined organic extracts were washed with H_2_O (3×20 mL), dried over MgSO_4_ and concentrated under reduced pressure. Column chromatography [SiO_2_, hexane/EtOAc (4:1)] then gave the product boronate ester **17** as an amorphous solid (281 mg, 90%). *R_f_* [SiO_2_, hexane/EtOAc (4:1)]: 0.13. Mp: 135–137 °C. *ν*_max_ (ATR): 2982 (CH), 2361 (CN), 1614 (Ar) cm^−1^. *δ*_H_ (400 MHz, CDCl_3_): 7.84 (2H, d, *J*=7.1 Hz, H-2 and H-6), 7.32 (2H, d, *J*=7.2 Hz, H-3 and H-5), 3.94 [1H, t, *J*=6.8 Hz, CH(CN)_2_], 3.26 (2H, d, *J*=6.8 Hz, CH_2_), 1.34 (12H, s, 4×CH_3_). *δ*_C_ (100 MHz, CDCl_3_): 135.86 (C), 135.61 (CH), 128.46 (CH), 112.19 (C), 84.00 (C), 77.30 (CH), 36.66 (CH_2_), 24.85 (CH_3_). LRMS (CI^+^): 283 [(M+H)^+^, 100%]. HRMS: 283.1613. C_16_H_20_O_2_N_2_^11^B requires (M+H)^+^, 283.1621.

### 2.9 2-Cyano-3-[4′-(4″,4″,5″,5″-tetramethyl-1″,3″,2″-dioxaborolan-2″-yl)phenyl]-2-(4‴-trifluoromethoxyphenyldiazo)propionitrile **19**

A solution of NaNO_2_ (25 mg, 0.36 mmol) in H_2_O (0.3 mL) was cooled to 0 °C and added dropwise to a stirred solution of 4-trifluoromethoxyaniline (62.8 mg, 0.355 mmol) and concd HCl (0.2 mL) in H_2_O (1.5 mL) at 0 °C. The resulting solution of diazonium salt **18** was stirred for 5 min and then added dropwise to a stirred solution of boronate **17** (100 mg, 0.354 mmol) and NaOAc (116 mg, 1.42 mmol), in a mixture of H_2_O (1.3 mL), MeOH (1.5 mL) and EtOH (1.5 mL) at 0 °C. The resultant solution was stirred at 0 °C for 1 h and then at rt overnight. The precipitate was filtered off and washed with ice-cold H_2_O (4 mL) to yield the caged uncoupler **19** as an amorphous yellow solid. Further material was obtained by concentration of the filtrate under reduced pressure followed by extraction with DCM. Removal of solvent under reduced pressure followed by chromatography [SiO_2_, petroleum ether/EtOAc (9:1)] gave further caged uncoupler **19** (combined yield 77 mg, 46%). *R_f_* [SiO_2_, petroleum ether/EtOAc (9:1)]: 0.34. Mp: 120–122 °C. *ν*_max_ (ATR): 2937 (CH), 2162 (CN), 1614 (Ar) cm^−1^. *δ*_H_ (CDCl_3_, 400 MHz): 7.90 (2H, d, *J*=9.0 Hz, H-2‴ and H-6‴), 7.82 (2H, d, *J*=8.0 Hz, H-3′ and H-5′), 7.36–7.39 (4H, m, H-2′, H-6′, H-3‴ and H-5‴), 3.69 (2H, s, CH_2_), 1.34 (12H, s, 4×CH_3_). *δ*_C_ (CDCl_3_, 100 MHz): 153.08 (C), 147.71 (C), 135.51 (CH), 132.86 (C), 130.15 (CH), 125.81 (CH), 121.48 (CH), 120.38 (q, *J*=259.2 Hz), 111.85 (C), 84.17 (C), 69.35 (C), 36.62 (CH_2_), 24.99 (CH_3_). *δ*_F_ (CDCl_3_, 376 MHz): −57.58. LRMS (CI^+^): 471 [(M+H)^+^, ^11^B, 11%], 446 (39), 337 (40), 283 (95), 219 (91), 189 (100). HRMS: 471.1821. C_23_H_23_O_3_N_4_F_3_^11^B requires (M+H)^+^, 471.1820.

### 2.10 Details of kinetic experiments

The stock solutions of caged uncouplers were made up by weight in DMF (caged uncoupler **15**, 1 mM; caged uncoupler **19**, 10 mM). The concentration of the stock H_2_O_2_ solution was checked by titration against KMnO_4_,^25^ and diluted accurately to a concentration of 1 mM. Reactions were carried out in a 1:1 mixture of DMF and 0.14 M aqueous sodium bicarbonate (pH 8.3).

Absorption measurements were made on a JASCO V550 double beam spectrophotometer using matched quartz cuvettes of 1 cm pathlength, with the cuvette compartment maintained at 37 °C by a circulating water bath. The spectrophotometer was calibrated using a solution of potassium chromate in 0.05 M KOH, whose absorption coefficient at 372 nm is 4830 M^−1^ cm^−1^.^26^ For this standard solution, the measured absorbance was proportional to concentration up to an absorbance of 1.2; all measurements reported in this paper were made with absorbance values less than 1.0.

Comparison between the spectra of caged uncoupler **15** and DNP^−^ **3**, and between caged uncoupler **19** and FCCP^−^ **4** showed that the appropriate wavelengths to monitor the reactions were 410 nm and 385 nm, respectively, since in each case the starting material showed a very much smaller absorption than the product. The appropriate absorption coefficients for the products (DNP^−^ **3** at 410 nm and FCCP^−^ **4** at 385 nm) under the conditions used for the reactions were determined using a parallel dilution approach in which stock solutions of these compounds were diluted into buffer systems where the absorption coefficients had been published,^27,28^ and into the buffer system used in the present work. Comparison of the observed absorbance values from these parallel dilutions allowed the appropriate coefficients for our experiments to be calculated (20,500 M^−1^ cm^−1^ at 410 nm for DNP^−^ **3** and 29,700 M^−1^ cm^−1^ at 385 nm for FCCP^−^ **4**).

Reactions were initiated by adding small aliquots of H_2_O_2_ to solutions of caged uncoupler **15** or caged uncoupler **19** in the DMF/buffer mixture, in a total volume of 1 mL. The blank reaction contained DMF/buffer in place of the H_2_O_2_. Control experiments showed that, under the conditions used, there was a slow breakdown of the caged uncouplers in the buffer; in the case of caged uncoupler **15**, this amounted to 3% of the initial compound over a time period of 2000 s; in the case of caged uncoupler **19**, this amounted to 14% of the initial compound over a time period of 1000 s. Since the reactions were carried out with the caged uncouplers in considerable excess over H_2_O_2_ and the uncouplers in DMF/buffer solution were used in the blank reaction, the spontaneous breakdown did not affect the observed *pseudo*-first order rate constants for the production of DNP^−^ **3** or FCCP^−^ **4** or the observed yields, although it would have small effects on the calculated second order rate constants. The concentrations of the caged uncouplers and H_2_O_2_ were chosen to give convenient rates of reaction while maintaining a reasonable approximation to *pseudo*-first order conditions in order to facilitate kinetic analysis. For caged uncoupler **15**, the concentration used was 200 μM and the H_2_O_2_ concentrations 10 μM and 20 μM. Reactions were performed in triplicate. For caged uncoupler **19**, the concentration used was 50 μM, and the H_2_O_2_ concentrations 5 μM and 10 μM. Reactions were performed in triplicate at 5 μM H_2_O_2_, and in duplicate at 10 μM H_2_O_2_. In each case, the experimental data (absorbance vs time) were fitted to a first order kinetic process using Microcal Origin software; this gave the end-point (limiting absorbance change) and the first order rate constant. Division of this rate constant by the concentration of the caged uncoupler in excess yielded the second order rate constant for the reaction.
